# Early IL-10 promotes vasculature-associated CD4^+^ T cells unable to control *Mycobacterium tuberculosis* infection

**DOI:** 10.1172/jci.insight.150060

**Published:** 2021-11-08

**Authors:** Catarina M. Ferreira, Ana Margarida Barbosa, Palmira Barreira-Silva, Ricardo Silvestre, Cristina Cunha, Agostinho Carvalho, Fernando Rodrigues, Margarida Correia-Neves, António G. Castro, Egídio Torrado

**Affiliations:** Life and Health Sciences Research Institute (ICVS), School of Medicine, University of Minho, Braga, Portugal. ICVS/3B’s - PT Government Associate Laboratory, Braga/Guimarães, Portugal.

**Keywords:** Immunology, Infectious disease, Adaptive immunity, Cytokines, Tuberculosis

## Abstract

Cytokine-producing CD4^+^ T cells play a crucial role in the control of *Mycobacterium tuberculosis* infection; however, there is a delayed appearance of effector T cells in the lungs following aerosol infection. The immunomodulatory cytokine IL-10 antagonizes control of *M*. *tuberculosis* infection through mechanisms associated with reduced CD4^+^ T cell responses. Here, we show that IL-10 overexpression only before the onset of the T cell response impaired control of *M*. *tuberculosis* growth; during chronic infection, IL-10 overexpression reduced the CD4^+^ T cell response without affecting the outcome of infection. IL-10 overexpression early during infection did not, we found, significantly impair the kinetics of CD4^+^ T cell priming and effector differentiation. However, CD4^+^ T cells primed and differentiated in an IL-10–enriched environment displayed reduced expression of CXCR3 and, because they did not migrate into the lung parenchyma, their ability to control infection was limited. Importantly, these CD4^+^ T cells maintained their vasculature phenotype and were unable to control infection, even after adoptive transfer into low IL-10 settings. Together our data support a model wherein, during *M*. *tuberculosis* infection, IL-10 acts intrinsically on T cells, impairing their parenchymal migratory capacity and ability to engage with infected phagocytic cells, thereby impeding control of infection.

## Introduction

Tuberculosis (TB) remains the worldwide leading cause of death from a single infectious agent. While active intervention is lowering the global incidence of this disease, new tools are required to reach the WHO’s ambitious goal of ending TB epidemics by 2035. These tools include improved diagnostics of clinical and subclinical disease and more effective drugs and vaccines (1). Specifically, a more effective vaccination regimen would make a crucial impact to interrupt TB transmission and control TB epidemics (1). Indeed, the current TB vaccine *Mycobacterium bovis* bacille Calmette-Guérin (BCG), while effective in preventing disseminated forms of pediatric TB (2), is not efficient against adult pulmonary disease (3, 4). Therefore, a novel vaccine regimen to protect against pulmonary TB is among the highest global health priorities.

The rational design of more effective vaccines requires a clear understanding of the immunological mechanisms of protection against pulmonary TB. In this regard, a substantial number of data support IFN-γ–producing CD4^+^ T cells (Th1 cells) as appropriate targets for novel vaccines because these cells are critical to TB control (5–9). Therefore, over the last decade significant efforts have gone into developing new vaccine regimens that elicit strong IFN-γ–producing CD4^+^ T cell responses or that boost BCG-induced IFN-γ–producing CD4^+^ T cells. However, the IFN-γ response is not a reliable correlate of protection against TB in humans (10–12). Accordingly, in a recent efficacy trial of modified vaccinia virus Ankara expressing Ag85A from *M*. *tuberculosis*, the Th1-boosting TB vaccine candidate failed to improve protection in BCG-vaccinated infants (13). While novel concepts for TB vaccination are emerging (14), these data suggest that the rational design of novel TB vaccines relying on cytokine-producing CD4^+^ T cells requires a better understanding of the crucial components of an effective CD4^+^ T cell response against *M*. *tuberculosis* infection.

Recent data show that different populations of CD4^+^ T cells develop in the lungs following *M*. *tuberculosis* infection (15). CD4^+^ T cells expressing programmed cell death 1 protein (PD-1) are a self-renewing population that produce low levels of IFN-γ whereas CD4^+^ T cells expressing the killer cell lectin-like receptor G1 (KLRG1) are short-lived but produce high levels of IFN-γ (15). Crucially, adoptive transfer experiments have unraveled a critical role of PD-1–expressing CD4^+^ T cells, but not KLRG1-expressing CD4^+^ T cells, in the control of *M*. *tuberculosis* growth (16). Moreover, while PD-1 cells express CXCR3 and locate within the lung parenchyma, KLRG1-expressing cells coexpress CX3CR1 and locate in the lung vasculature (16–18). Indeed, KLRG1-expressing cells cuff around blood vessels and do not engage with infected macrophages in the parenchyma, which is critical for the protective function of CD4^+^ T cells (19). Taken together, these data show that the parenchymal migratory capacity and the ability to engage with infected phagocytes within the lung lesions are critical features of protective T cells. Therefore, it is critical to identify the factors impeding the rapid migration of CD4^+^ T cells into the lung parenchyma. This will lead to the rational design of vaccines that generate T cells capable of entering the lung lesion and thereby mediate long-lasting protection against TB.

IL-10 has been implicated in increased susceptibility to TB, both in humans and in animal models, an event highly associated with decreased CD4^+^ T cell responses (20–26). In humans, IL-10 is elevated in the pleural fluid (27), bronchoalveolar lavage (28), sputum (29), and serum (30, 31) of patients with active TB compared with healthy controls. Mechanistically, IL-10 impairs the proliferation of IFN-γ–producing T cells stimulated with *M*. *tuberculosis* (22, 23). These observations are supported by mouse data wherein the absence of IL-10 in the genetically resistant strains C57BL/6 and BALB/c enhanced the influx of CD4^+^ T cells into the lungs, resulting in elevated production of T cell–recruiting chemokines and protective cytokines, including IFN-γ, TNF, and GM-CSF (32). Furthermore, the genetically susceptible CBA/J mouse line, and transgenic lines overexpressing IL-10, display enhanced bacterial burdens associated with reduced Th1 responses and impaired macrophage bactericidal functions (24, 25, 33). These data taken together demonstrate that IL-10 has an antagonistic role in the control of *M*. *tuberculosis* infection; however, the immunological consequences of IL-10 production during TB are currently unknown. Crucially, recent data show that IL-10 blockade during BCG vaccination and following *M*. *tuberculosis* infection enhances Ag-specific responses and provides significantly greater protection against aerogenic *M*. *tuberculosis* challenge (34). Therefore, in this work we used a novel mouse model of controlled IL-10 overexpression recently described (pMT-10) (35) to define the temporal role and the mechanisms whereby this cytokine modulates protective immunity to TB. Using this model, IL-10 overexpression was induced in pMT-10 mice by feeding the mice a 2% sucrose solution with 50 mM of zinc sulfate (35).

Our data show that IL-10 overexpression impairs the protective response, prompting uncontrolled bacterial proliferation and severe immunopathological consequences to the host, during the early stages of *M*. *tuberculosis* infection but not during chronic infection. During the early stages of infection, IL-10 overexpression altered neither populations of antigen-presenting cells (APCs) in the mediastinal lymph nodes (MLNs) nor the kinetics of CD4^+^ T cell activation. Instead, CD4^+^ T cells primed in the IL-10–enriched environment were recruited to the lungs but accumulated in the lung vasculature and did not translocate into the parenchyma. This migratory deficit prevented antigen sensing, which limited proliferation and effector cytokine production. Crucially, in adoptive transfer from the lungs of mice overexpressing IL-10 into RAG^–/–^ mice, effector CD4^+^ T cells primed in the IL-10–enriched environment maintained their vasculature phenotype and were unable to restrain *M*. *tuberculosis* infection. Together our data support a model wherein IL-10 compromises the protective function of CD4^+^ T cells by promoting the differentiation of a vasculature-associated phenotype with reduced ability to translocate into the lung parenchyma and infiltrate the infected lesion.

## Results

### Early but not late IL-10 production impairs the control of airborne M. tuberculosis infection.

The progression and severity of TB has been associated with increased levels of IL-10 in both humans (27, 28, 30, 31, 36) and mice (24, 25, 33). Despite this, the immunological consequences of IL-10 overexpression following aerogenic *M*. *tuberculosis* infection have not been fully defined. We used the recently described pMT-10 transgenic mouse line (35) wherein IL-10 overexpression can be induced at different stages of infection by feeding the mice a 2% sucrose solution with 50 mM zinc sulfate. Inducing IL-10 overexpression allowed us to define the temporal role and mechanisms by which IL-10 antagonizes the protective immune response and control of *M*. *tuberculosis* infection. After aerogenically infecting pMT-10 mice with *M*. *tuberculosis*, in one group we induced IL-10 overexpression starting at day 5 after infection to determine the early-phase impact of this cytokine in the ability of mice to control *M*. *tuberculosis* bacterial burdens. To determine the cytokine’s chronic-phase impact, in another group we induced IL-10 overexpression starting at day 30 after infection. B6 mice used as controls were also fed with the 2% sucrose solution with 50 mM zinc sulfate used to induce IL-10 overexpression in pMT-10 mice.

**Figure 1:**
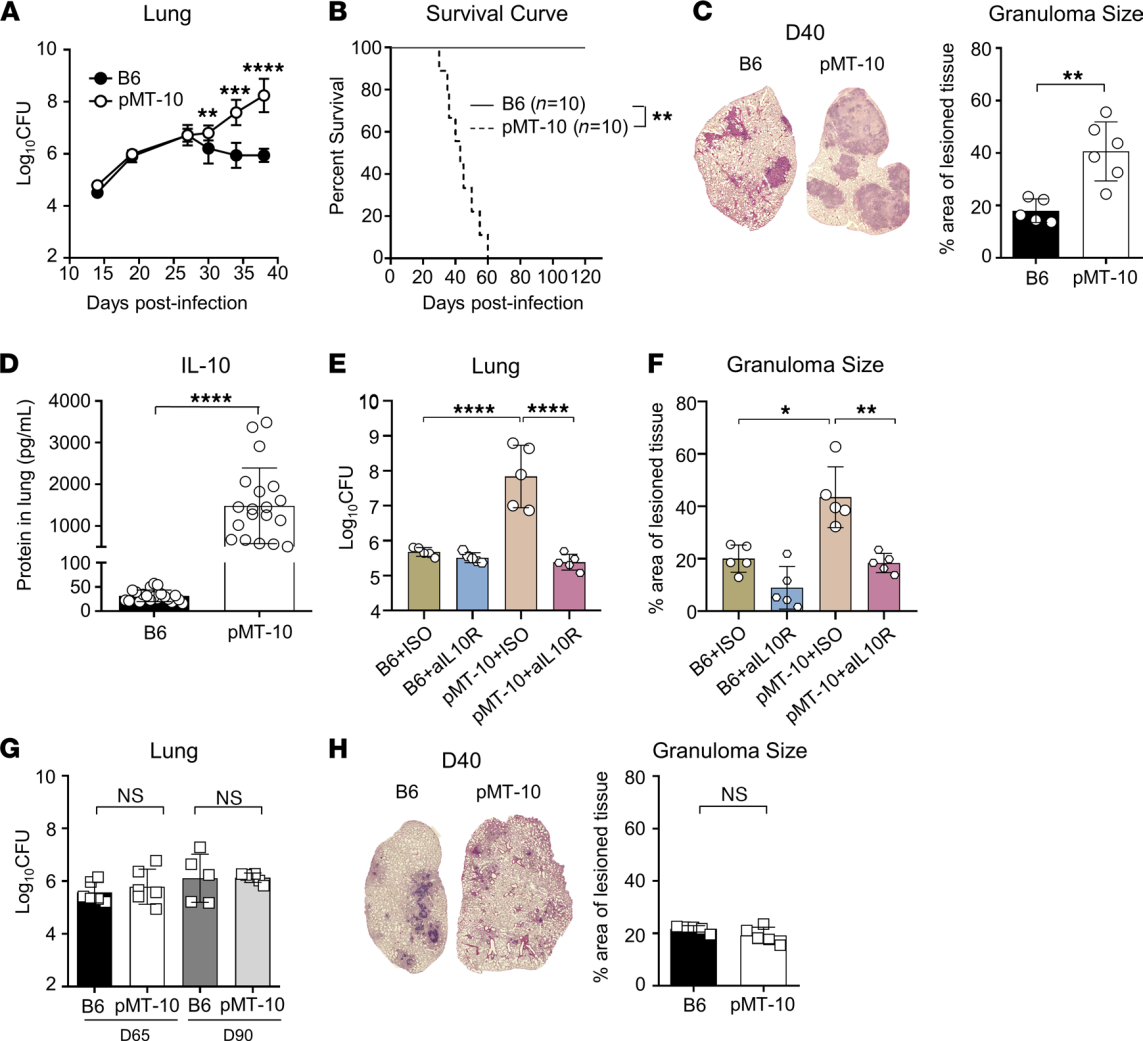
Early, but not late, IL-10 overexpression affects the outcome of aerogenic *M. tuberculosis* infection. B6 and pMT-10 mice were infected with *M*. *tuberculosis* H37Rv via aerosol route and IL-10 overexpression induced after day 5 after infection (**A–F**). (**A**) Lung bacterial burdens and (**B**) survival of B6 and pMT-10 mice following *M*. *tuberculosis* infection. (**C**) Representative hematoxylin and eosin–stained lung sections and percentage of infiltrated area in the lungs at day 40 after infection. Individual data points represent individual animals. Lung bacterial burdens and survival and histology data are representative of 3 independent experiments with 4–5 mice per group. (**D**) IL-10 concentration in lung supernatants at day 30 after infection, determined by ELISA. Data represent a composite of 5 independent experiments with 4–5 mice per group. **P < 0.01; ***P < 0.001; ***P < 0.0001 using Student’s *t* test. (**E**) Lung bacterial burden and (**F**) percentage of lung infiltrated area at day 40 after infection in B6 and pMT-10 mice that were injected weekly with an anti–IL-10R or control Ab starting 1 day before infection. (**E **and** F**) Data are representative of at least 3 independent experiments with 5 mice per group. **P < 0.01 by 1-way ANOVA followed by Tukey’s test. B6 and pMT-10 mice were infected with *M*. *tuberculosis* H37Rv via aerosol route and IL-10 overexpression induced at day 30 after infection (**G** and **H**). (**G**) Lung bacterial burden of B6 and pMT-10 mice at days 65 and 90 after infection. (**H**) Representative hematoxylin and eosin–stained lung sections and percentage of infiltrated area in lungs at 65 days after infection. Individual data points represent individual animals. Data represent 3 independent experiments with 4–5 mice per group. Data area shown as the mean ± SD. **P < 0.01; ***P < 0.001; ***P < 0.0001 by Student’s *t* test.

We began by testing the impact of IL-10 overexpression during the early stages of infection. After aerosol infection, we found similar bacterial burdens in both groups of mice until day 27 after infection (Figure 1A). While B6 mice controlled *M*. *tuberculosis* growth from this point onward, bacterial growth continued to increase in pMT-10 mice (Figure 1A). Consequently, pMT-10 mice displayed significantly lower survival compared with B6 mice (Figure 1B). Gross histological examination revealed that, shortly before they succumbed to infection, there were larger lesions in the lungs of pMT-10 mice than in B6 mice (Figure 1C). As expected, the increased susceptibility of pMT-10 mice was associated with increased IL-10 levels in the lungs (Figure 1D), and the blockade of IL-10R activity reverted the susceptible phenotype of pMT-10 mice that now displayed similar bacterial burdens (Figure 1E) and lesion sizes compared with B6 mice (Figure 1F).

We next sought to determine the impact of IL-10 overexpression during the chronic phase of *M*. *tuberculosis* infection. To do this, we quantified bacterial loads after inducing IL-10 overexpression from day 30 to day 60 or 90 after infection. Compared with B6 controls, pMT-10 mice showed no marked differences in lung bacterial loads (Figure 1G) or lung pathology (Figure 1H).

Taken together, these data showed that IL-10 overexpression during the early stages of the *M*. *tuberculosis* infection promoted progression of infection and resulted in the hosts’ rapid death. On the other hand, IL-10 overexpression induced during the chronic phase of infection significantly altered neither progression nor control of *M*. *tuberculosis*.

### Early IL-10 production delays the accumulation of IFN-γ–producing CD4^+^ T cells in the lungs.

**Figure 2:**
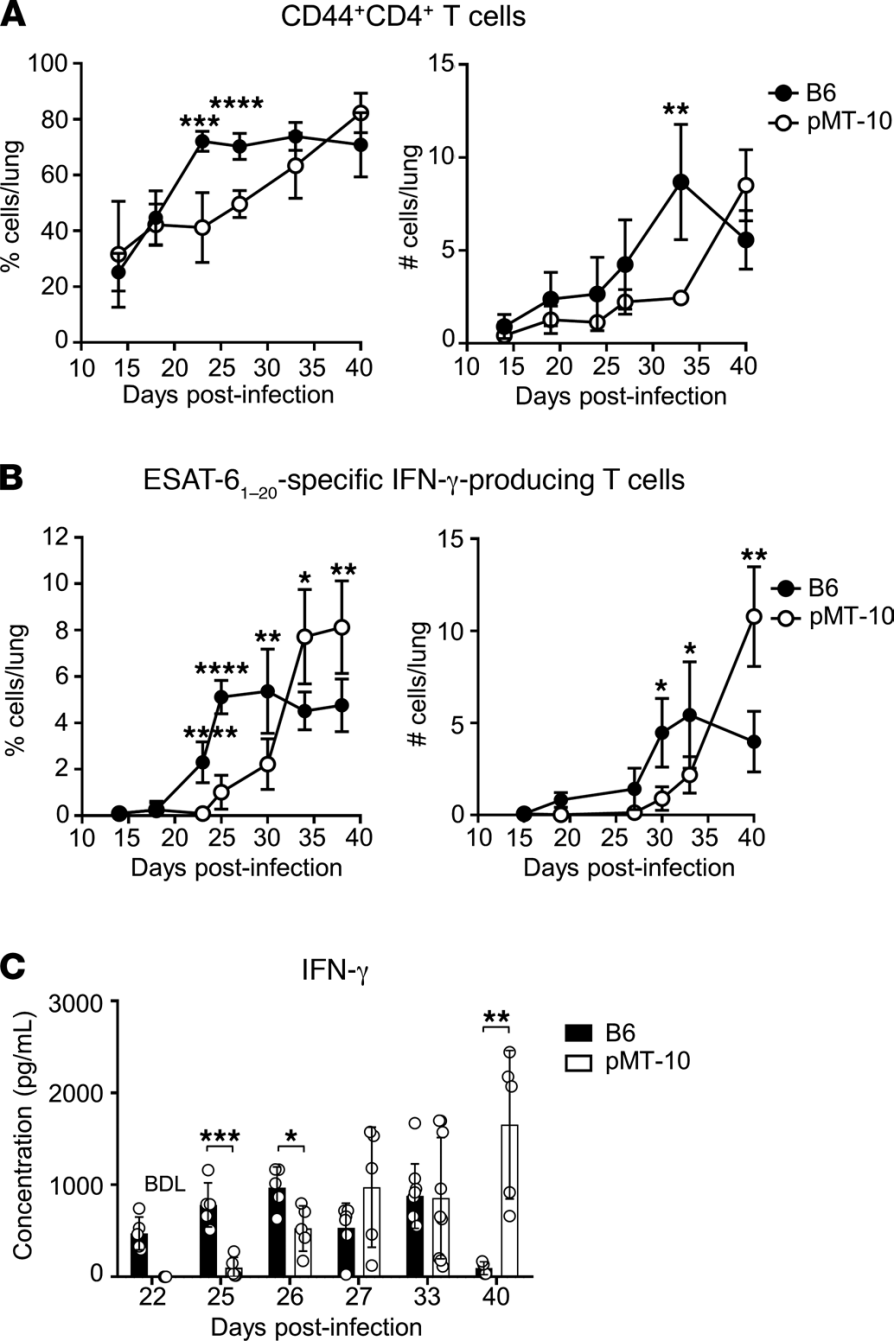
IL-10 overexpression delays the onset of the protective immune CD4^+^ T cell response. B6 and pMT-10 mice were infected with *M*. *tuberculosis* H37Rv via aerosol route and IL-10 overexpression induced after day 5 after infection. B6 mice maintained in the same conditions were used as controls. (**A**) Frequency and number of CD4^+^ T cells in the lungs of mice throughout infection. (**B**) Frequency and number of IFN-γ–producing CD4^+^ T cells after in vitro restimulation with the immunodominant ESAT-6_1–20_ peptide. (**C**) IFN-γ protein in lung supernatants determined by ELISA. Data represent at least 3 independent experiments with 5 mice per group. *P < 0.05; **P < 0.01; ***P < 0.001; ***P < 0.0001 by Student’s *t* test. NS: not significant.

The above data warranted further detailed analysis of IL-10’s role during the early stages of *M*. *tuberculosis* infection. As IL-10–mediated susceptibility to *M*. *tuberculosis* infection had been associated with reduced Th1 responses in both humans and mice (22, 23, 26, 29, 32, 34), we first asked if *M*. *tuberculosis*–infected pMT-10 mice displayed impaired CD4^+^ T cell responses to *M*. *tuberculosis* infection. To do this, we infected mice and induced IL-10 overexpression starting at day 5 after infection (as above) and analyzed the kinetics of the CD4^+^ T cell response in the lungs. Compared with B6 mice, pMT-10 mice showed a delayed accumulation of activated CD4^+^ T cells expressing the activation marker CD44 in their lungs (Figure 2A). To further explore the impact of IL-10 environment in the Ag-specific IFN-γ response, we determined the frequency and number of *M*. *tuberculosis*–specific CD4^+^ T cells and their ability to produce IFN-γ. To do this, we restimulated lung single-cell suspensions with the early-secreted antigenic target-6 (ESAT-6)_1–20_ epitope and determined IFN-γ producers by intracellular cytokine staining (18). In accordance with the above data, we found a delayed kinetics of *M*. *tuberculosis*–specific IFN-γ–producing CD4^+^ T cell accumulation in the lungs of pMT-10 mice compared with B6 mice (Figure 2B). Interestingly however, despite the high levels of IL-10, IFN-γ–producing CD4^+^ T cells increased rapidly in the lungs of pMT-10 mice after day 30 after infection. The kinetics of IFN-γ protein in the lungs of infected mice followed a similar pattern to that of the Ag-specific response determined by flow cytometry (Figure 2C). These data showed that IL-10 production early during infection would delay the accumulation of IFN-γ–producing CD4^+^ T cells required to control *M*. *tuberculosis* infection.

### IL-10 impairs the priming and effector differentiation of recently activated CD4^+^ T cells.

**Figure 3:**
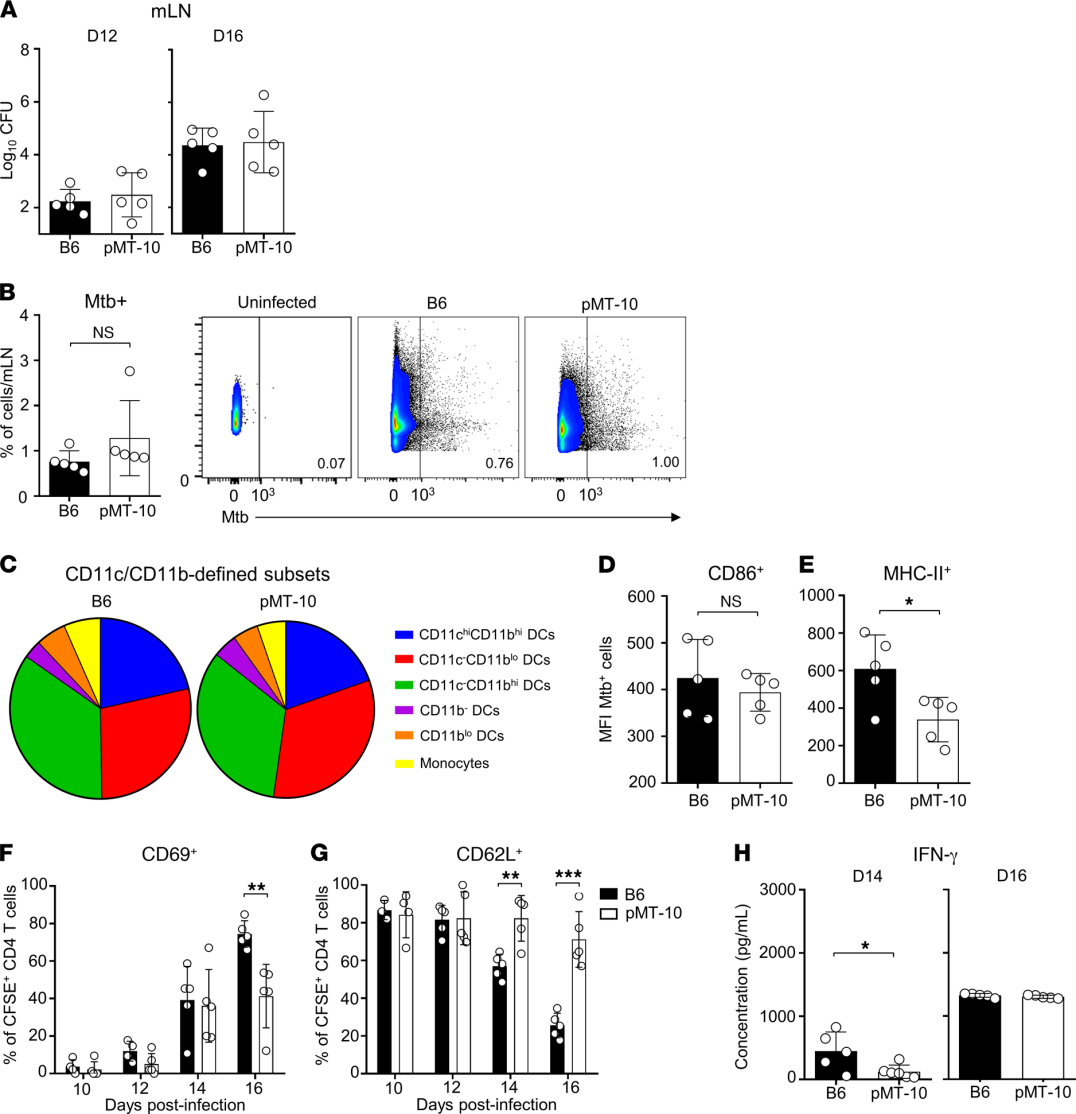
Early IL-10 overexpression does not significantly impair CD4^+^ T cell priming and differentiation in MLN. B6 and pMT-10 mice were infected via aerosol route with *M*. *tuberculosis*–mCherry and IL-10 overexpression induced after day 5 after infection. B6 mice maintained in the same conditions were used as controls. (**A**) MLN bacterial burdens at days 12 and 16 after infection. (**B**) Representative FACS plots and frequency of *M*. *tuberculosis*–mCherry–infected myeloid cells in the MLNs of B6 and pMT-10 mice at day 14 after infection. (**C**) Representative distribution of *M*. *tuberculosis*–mCherry-infected myeloid cell subsets in the MLNs of B6 and pMT-10 mice at day 14 after infection. (**D**) Mean fluorescence intensity of CD86 and (**E**) MHC-II in *M*. *tuberculosis*–mCherry-infected myeloid cells from the MLNs of B6 and pMT-10 mice at day 14 after infection. (**F**) Frequency of CFSE-labeled CD4^+^ T cells expressing CD69 and (**G**) CD62L at different points following aerosol infection with *M*. *tuberculosis*. (**H**) IFN-γ quantification in supernatants of ESAT-6_1–20_-stimulated single-cell suspensions prepared from lung-draining lymph nodes of B6 and pMT-10 mice infected for 14 or 16 days. Data represent 2 independent experiments with 4–6 mice per group. Data are shown as the mean ± SD. *P < 0.05; **P < 0.01; ***P < 0.001; ***P < 0.0001 using Student’s *t* test.

Reduced frequencies of Ag-specific CD4^+^ T cells in the lungs can result from defective priming of Ag-specific cells in the lung-draining lymph nodes or defective recruitment to the lungs. Taking into consideration that (a) *M*. *tuberculosis* dissemination to the MLNs is concomitant with CD4^+^ T cell priming (37, 38) and (b) IL-10 impairs dendritic cell trafficking to the MLNs (39), we first determined the impact of IL-10 in the transport of *M*. *tuberculosis* bacteria to the MLNs. To do this, we quantified the bacterial load in the MLNs at a time when CD4^+^ T cells would first encounter Ag (37). We found similar bacterial loads in B6 and pMT-10 MLNs at days 12 and 16 after infection, showing that IL-10 did not impair the transport of the bacteria from the lung to the MLN (Figure 3A). To determine if IL-10 expression altered the MLNs’ inflammatory context, we aerogenically infected B6 and pMT-10 mice with *M*. *tuberculosis*–mCherry and characterized *M*. *tuberculosis*–infected myeloid populations at day 14 after infection. For gating strategy, see Supplemental Figure 1; supplemental material available online with this article; https://doi.org/10.1172/jci.insight.150060DS1

We found that the MLNs from B6 and pMT-10 mice presented similar frequencies of infected cells (Figure 3B). Furthermore, the two strains’ profiles of infected cell populations were similar (Figure 3C), the majority of infected cells being CD11c^-^CD11b^hi^ and CD11c^-^CD11b^lo^. We additionally analyzed the expression of MHC-II and CD86 and found that, in *M*. *tuberculosis*–infected cells, IL-10 overexpression did not affect the expression of CD86 (Figure 3D) but it decreased the expression of MHC-II (Figure 3E). These data showed that IL-10 overexpression would not alter the profile of *M*. *tuberculosis*–infected cells within the MLNs of pMT-10 mice but it impaired MHC-II expression as previously described (40).

We next investigated if these effects of IL-10 had an impact in CD4^+^ T cell priming. To do this, we adoptively transferred CFSE-labeled P25 T cell receptor–transgenic cells (P25Tg, specific for Ag85b presented in the context of I-A^b^) into B6 or pMT-10 mice at different times after aerogenic *M*. *tuberculosis* infection and, 24 hours later, determined their activation status (CD69 and CD62L expression) in the MLN (37). We detected CD69 expression on similar proportions of cells transferred into B6 and pMT-10 mice, up to day 16 after infection, when there was a reduced expression of CD69 evident in P25Tg cells transferred into pMT-10 mice (Figure 3F). We also measured the expression of CD62L in transferred cells and found that, in B6 mice, transferred P25Tg cells lost the expression of CD62L faster than in pMT-10 mice (Figure 3G). This suggested a delay in differentiation of newly activated CD4^+^ T cells due to the IL-10 environment.

To clarify this issue, we restimulated MLN cells from B6 and pMT-10 mice with ESAT-6_1–20 _to measure IFN-γ production by Ag-specific CD4^+^ T cells. We found that, at day 14 after infection, there was a reduced production of IFN-γ by MLN cells in pMT-10 mice compared with B6 mice (Figure 3H). Nevertheless, at day 16 after infection, the levels of IFN-γ production were similar in B6 and pMT-10 (Figure 3H).

Together, these data suggested that IL-10 would not significantly impair the kinetics of CD4^+^ T cell priming although it would delay the differentiation of newly activated CD4^+^ T cells. Nevertheless, it was unlikely that the delay differentiation before day 16 after infection accounted for the delayed accumulation of IFN-γ–producing CD4^+^ T cells in the lungs (Figure 2).

**Figure 4:**
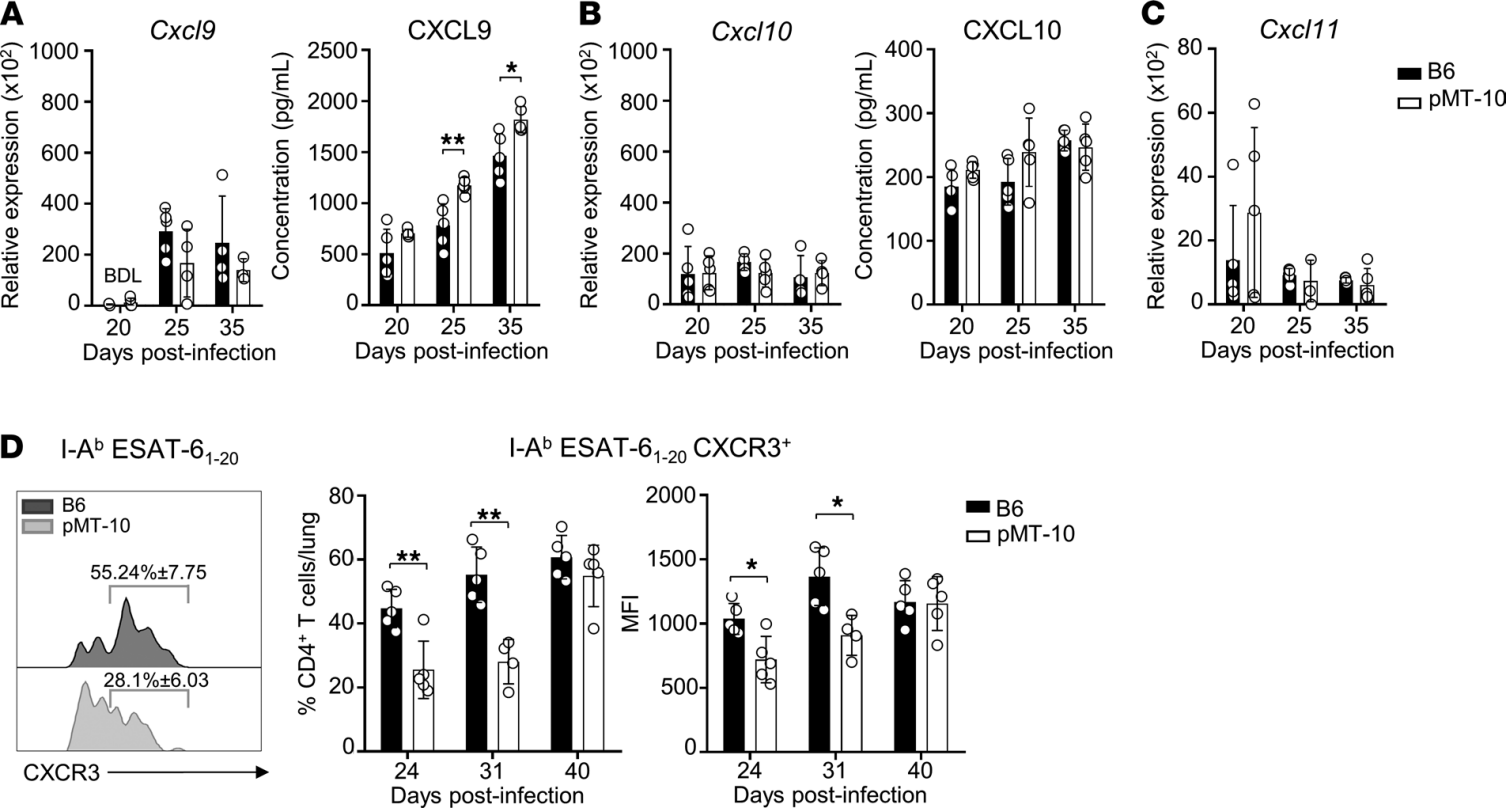
IL-10 overexpression does not impair chemokine production in *M. tuberculosis*–infected lungs. B6 and pMT-10 mice were infected with *M*. *tuberculosis* H37Rv via aerosol route and IL-10 overexpression induced after day 5 after infection. (**A**) Relative expression and protein levels of CXCL9, (**B**) CXCL10, and (**C**) CXCL11 in the lungs of infected mice. Protein levels of CXCL11 were below detection limit at every time point assessed. (**D**) Frequency of CXCR3-expressing I-A^b^ ESAT-6_1–20_–specific CD4^+^ T cells in the lungs of infected mice over the course of *M*. *tuberculosis* infection. Data represent 2 independent experiments with 4–5 mice per group. Data are shown as the mean ± SD. *P < 0.05; **P < 0.01; ***P < 0.001; ***P < 0.0001 using Student’s *t* test.

Recent data suggested that IL-10 may inhibit the expression of chemokines that guide Th1 cells to *M*. *tuberculosis* infection sites (32). Therefore, we next asked if IL-10 downregulated the production of chemokines that guide newly differentiated cells to the infected lungs. We quantified CXCL9, CXCL10, and CXCL11 in the lungs of infected mice and found similar protein and expression levels of these chemokines in B6 and pMT-10 mice (Figure 4, A–C). Previous data showed that, in the absence of IL-10, there was an upregulation of CXCL10 production in the lungs of *M*. *tuberculosis*–infected mice (32). However, the high levels of IL-10 in our model did not impair chemokine production in the lungs following *M*. *tuberculosis* infection. This response was likely a consequence of the elevated bacterial loads seen in our model. Indeed, we saw enhanced CXCL9 responses in pMT-10 mice after day 25 of infection, the time at which these mice begin displaying higher *M*. *tuberculosis* loads than B6 mice. As such, the data showed that the delayed accumulation of IFN-γ–producing CD4^+^ T cells was not caused by altered chemokine expression in the lungs.

We then asked if Ag-specific CD4^+^ T cells from B6 and pMT-10 mice would express similar levels of CXCR3, the chemokine receptor for CXCL9, CXCL10, and CXCL11. Compared with B6 mice, we found in pMT-10 mice that ESAT-6_1–20_–specific CD4^+^ T cells from the lungs displayed reduced expression of CXCR3 at days 24 and 31 after infection (Figure 4D). Together, these data showed that IL-10 would not impair the production of chemokines that guided CD4^+^ T cells to the *M*. *tuberculosis*–infected lungs but it would inhibit the expression of chemokine receptors essential for CD4^+^ T cells to enter the lung parenchyma and induce control of infection (16, 41).

### IL-10 impairs the migration of CD4^+^ T cells into the lung parenchyma.

**Figure 5:**
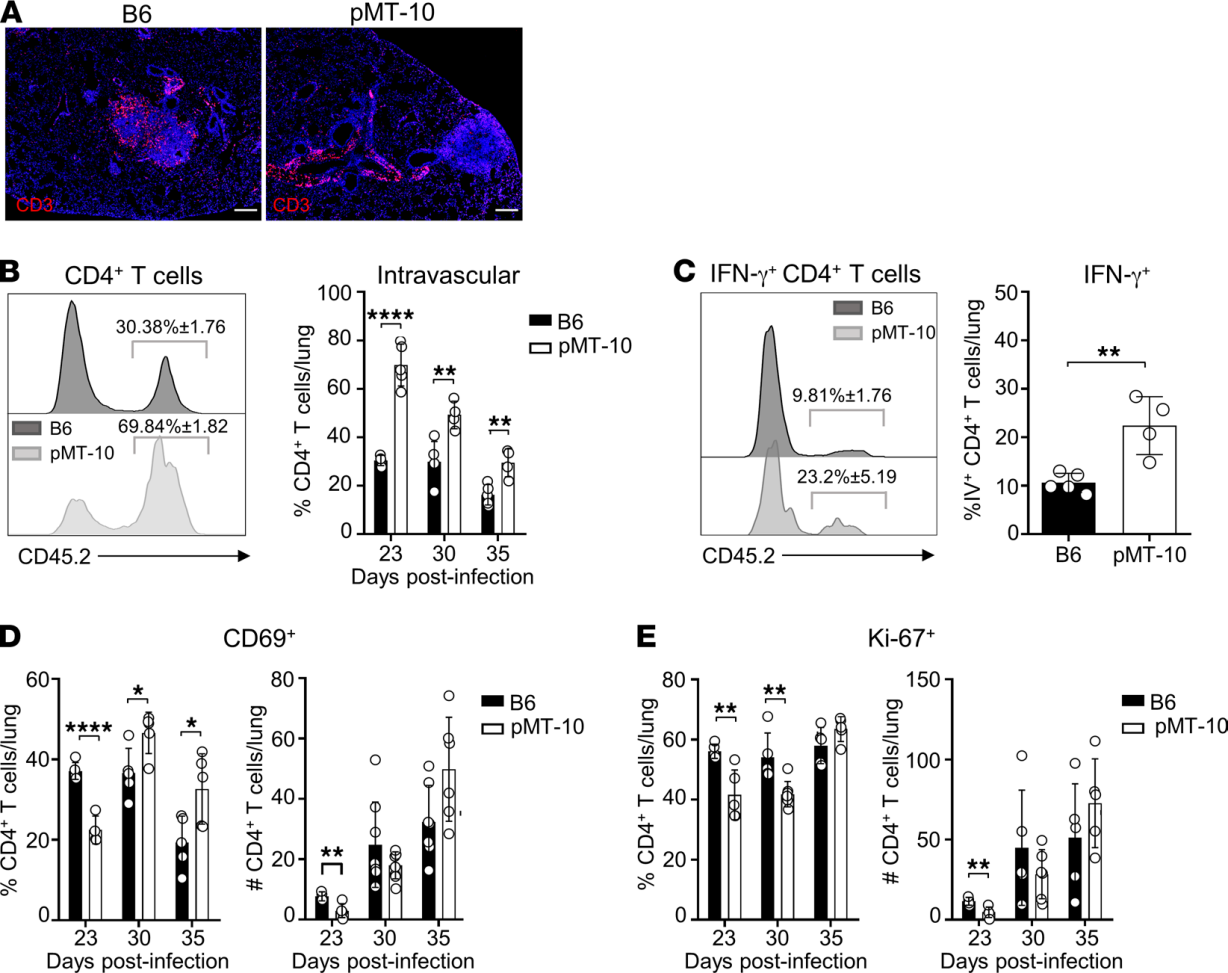
IL-10 overexpression limits CD4^+^ T cell migration into lung parenchyma and impairs antigen recognition and proliferation. B6 and pMT-10 mice were infected with *M*. *tuberculosis* H37Rv via aerosol route and IL-10 overexpression induced after day 5 after infection. (**A**) Representative immunofluorescence of CD3^+^ cells in lungs of mice at day 30 after infection. Scale bar: 200 μm. (**B**) Flow cytometry analysis and frequency of intravascular (CD45^+^) CD4^+^ T cells in lungs of mice throughout infection. (**C**) Flow cytometry analysis and frequency of intravascular (CD45^+^) IFN-γ–producing CD4^+^ T cells in lungs of mice at day 30 after infection. (**D**) Frequencies and numbers of CD69^+^CD4^+^ T cells from the lungs of mice throughout infection. (**E**) Frequencies and numbers of Ki-67^+^CD4^+^ T cells from lungs of mice throughout infection. Data represent 2 independent experiments with 4–5 mice per group. Data are shown as the mean ± SD. *P < 0.05; **P < 0.01; ***P < 0.001; ***P < 0.0001 using Student’s *t* test.

Our results showed that *M*. *tuberculosis*–specific CD4^+^ T cells present in the lungs of pMT-10 mice expressed reduced levels of CXCR3. We therefore hypothesized that IL-10 would antagonize control of *M*. *tuberculosis* infection by impairing the translocation of CD4^+^ T cells into the lung parenchyma, where they could interact with infected phagocytes to induce control of infection. In accordance with this hypothesis, the histological examination of lung sections from *M. tuberculosis*–infected mice 30 days after infection revealed the formation of perivascular cuffs in pMT-10 mice whereas in B6 mice lymphocytes localized within the granuloma (Figure 5A). Therefore, we performed intravital flow cytometry to determine the location at days 23, 27, and 33 after infection of CD4^+^ T cells in the lungs of B6 and pMT-10 mice. To do this, we intravenously injected mice with a fluorochrome-labeled CD45.2 antibody, and mice were culled 3 minutes after injection. Lungs were then collected and processed for flow cytometry analysis as previously described (16). Our results showed that pMT-10 mice presented increased frequencies of intravascular CD4^+^ T cells at all time points analyzed (Figure 5B). They also presented intravascular CD4^+^ T cells capable of producing IFN-γ in response to ESAT-6_1–20_ at day 33 after infection (Figure 5C).

**Figure 6:**
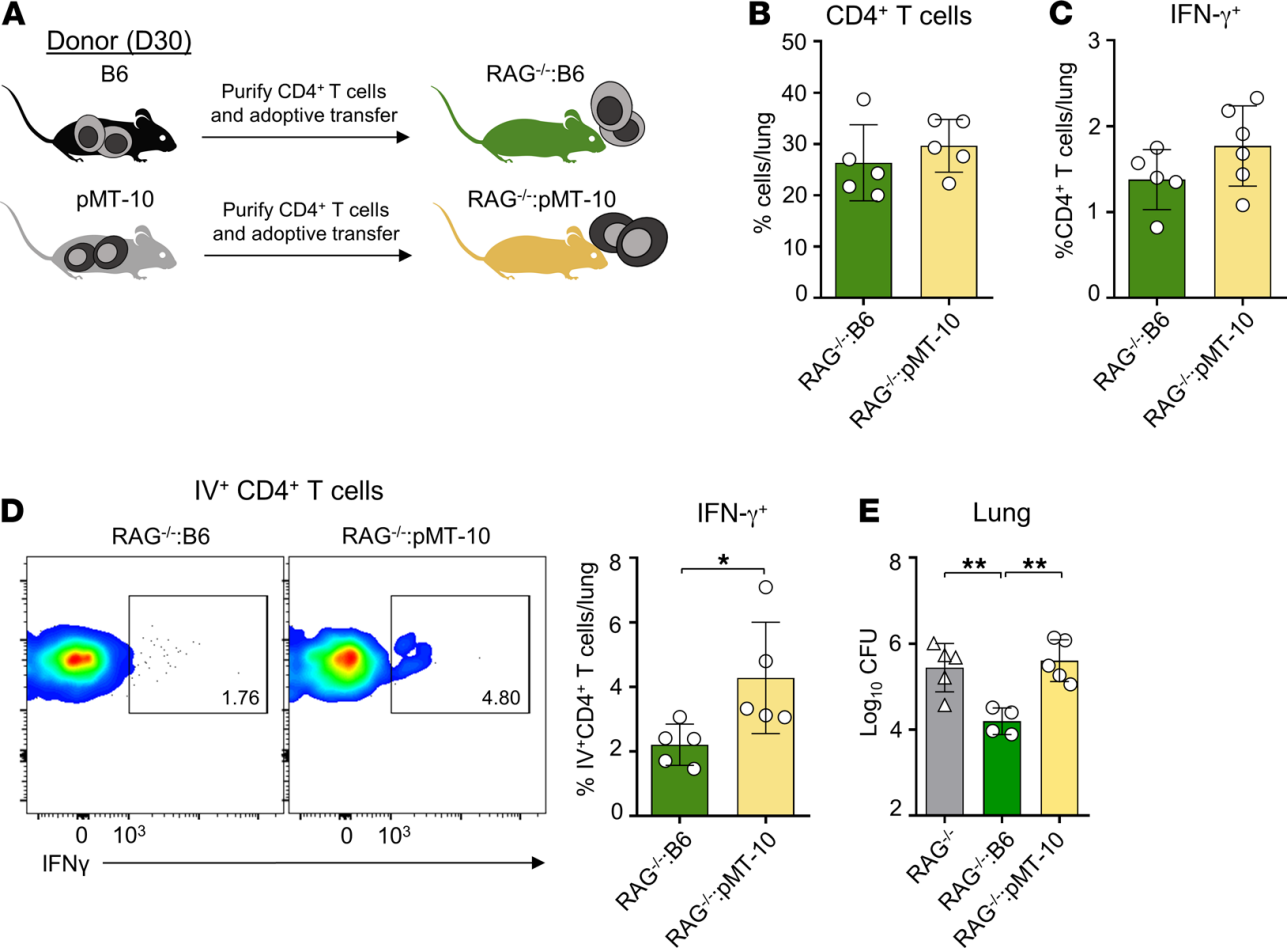
CD4^+^ T cells differentiated in IL-10–rich environment maintain their vasculature phenotype in an environment with normal levels of IL-10. (**A**) B6 and pMT-10 mice were infected with *M*. *tuberculosis* H37Rv via aerosol route and IL-10 overexpression was induced after day 5 after infection. At day 30 after infection, B6 and pMT-10 mice were sacrificed and 5 × 10^5^ CD4^+^ T cells, purified from their lungs, were adoptively transferred into RAG-deficient mice infected 15 days earlier. Frequencies of (**B**) CD4^+^ T cells and (**C**) CD4^+^ T cells capable of producing IFN-γ in response to ESAT-6_1–20_ at day 15 after adoptive transfer in lungs of infected mice. (**D**) Representative flow cytometry analysis and frequencies of CD4^+^ T cells capable of producing IFN-γ in response to ESAT-6_1–20_ in lung vasculature of infected mice. Each flow plot represents 1 animal per group. (**E**) Lung bacterial burdens in RAG^–/–^ mice that received B6 or pMT-10 effector cells at day 30 after infection (15 days after adoptive transfer). Data represent 2 independent experiments with 4–5 mice per group. Data are shown as the mean ± SD. *P < 0.05; **P < 0.01 using Student’s *t* test.

The reduced migration of CD4^+^ T cells to the lung parenchyma of pMT-10 mice was associated with reduced frequencies of CD4^+^ T cells expressing the early activation marker CD69 at day 23 after infection (Figure 5D). It was also associated with reduced proliferation of CD4^+^ T cells, as determined by the expression of Ki-67, until day 30 after infection (Figure 5E). Nevertheless, from day 30 after infection onward, pMT-10 mice displayed an increase in the frequency and number of CD4^+^ T cells expressing CD69 (Figure 5D) and Ki-67 (Figure 5E). This increase was associated with the increased Ag-specific IFN-γ response described above (Figure 2C). Despite this increase, bacterial growth was not curbed by the antigen-specific IFN-γ response. Therefore, we questioned the ability of effector CD4^+^ T cells generated in a high IL-10 environment to control *M*. *tuberculosis* infection. To measure this ability, we purified CD4^+^ T cells from the lungs of B6 or pMT-10 mice at day 30 after infection and adoptively transferred them intravenously into recipient RAG^–/–^ mice infected for 15 days. We then analyzed the accumulation of these cells in the vasculature and their ability to control infection (Figure 6A).

At day 30 after infection, we confirmed that RAG^–/–^:B6 and RAG^–/–^:pMT-10 mice presented similar frequencies of total CD4^+^ T cells (Figure 6B) and total *M*. *tuberculosis*–specific IFN-γ–producing CD4^+^ T cells (Figure 6C) in the lungs. Interestingly, however, we found increased frequencies of intravascular IFN-γ–producing CD4^+^ T cells in the lungs of recipient mice populated by pMT-10 CD4^+^ T cells (Figure 6D). These data showed the intrinsic impact of IL-10 in CD4^+^ T cells’ ability to locate within the lungs, independent of the lung microenvironment. Importantly, we found that this phenotype was associated with increased lung bacterial burdens (Figure 6E), highlighting the association between CD4^+^ T cells’ defective spatial distribution in the lungs and susceptibility to infection.

Overall, our data supported a model wherein IL-10 would have a detrimental impact in the development of the acquired immune response. This effect would be mediated by the differentiation of CD4^+^ T cells that display a vasculature phenotype unable to interact with infected phagocytes to induce control of *M*. *tuberculosis* growth.

## Discussion

Although IL-10 has been associated with increased susceptibility to *M*. *tuberculosis* infection in humans (27, 28, 30, 31, 36) and in mice (24, 25, 39), the immunological mechanisms underlying this effect are not completely understood. According to the data we obtained in this study, IL-10 overexpression during the early stages of the *M*. *tuberculosis* infection promotes uncontrolled bacterial proliferation in the lungs and has severe immunopathological consequences as well as reduced survival of the host. In stark contrast with these data, IL-10 overexpression after the onset of the T cell response did not impact the outcome of infection. During the early stages of infection, IL-10 overexpression altered neither DC populations in MLN nor the kinetics of CD4^+^ T cell activation. Instead, CD4^+^ T cells primed in the IL-10–enriched environment were recruited to the lungs but accumulated in the vasculature and did not migrate into the parenchyma. This migratory deficit impaired antigen sensing, which limited the proliferation and effector cytokine production of recently activated effector CD4^+^ T cells. Crucially, the adoptive transfer of CD4^+^ T cells from the lungs of *M*. *tuberculosis*–infected mice into RAG^–/–^ mice revealed that CD4^+^ T cells primed and differentiated in the IL-10–enriched environment maintain their vasculature-associated phenotype and are unable to restrain *M*. *tuberculosis* infection. Together our data support a model wherein IL-10 compromises the protective function of CD4^+^ T cells by promoting the differentiation of a vasculature-associated phenotype with reduced ability to translocate into the lung parenchyma and infiltrate the infected lesion.

Previous published data show that IL-10 inhibits cytokine production by monocytes (42) and macrophages (43) while downregulating MHC-II expression (40, 42). These effects of IL-10 are consistent with the reduced Th1 responses following *M*. *tuberculosis* infection observed in genetic (24, 26) and transgenic murine models of IL-10 overexpression (25). Our data show that this reduced Th1 response is a consequence of IL-10 production during the onset of the T cell response. This is consistent with a recent study showing that blockade of IL-10R signaling during the first 20 days after aerogenic *M*. *tuberculosis* infection reverts susceptibility in CBA/J mice (26).

Moreover, our data show that CD4^+^ T cells generated in an IL-10–enriched environment display an impaired parenchymal migratory potential. Likely associated with the reduced expression of CXCR3, this impaired potential ultimately hampers CD4^+^ T cells’ protective function by compromising their ability to engage with infected phagocytes. Indeed, because T cells are better able to reduce bacterial growth if they are in direct contact with infected phagocytes (19), the parenchyma location of CD4^+^ T cells is crucial to control *M*. *tuberculosis* infection.

Early data on CXCR3^–/–^ mice suggested a redundant role for this chemokine receptor in the recruitment of CD4^+^ T cells to the lungs of *M*. *tuberculosis*–infected mice (44). However, more recent data show that CXCR3 is crucial for CD4^+^ T cells to populate the lung parenchyma and induce control of *M*. *tuberculosis* growth (16–18). Indeed, deficiency in this chemokine receptor decreases by half the rate at which Th1 cells enter the lung parenchyma (41). Therefore, the reduced expression of CXCR3 seen on Ag-specific CD4^+^ T cells from pMT-10 mice overexpressing IL-10 in early stages of infection likely plays a crucial role in the delayed migration of these cells into the lung parenchyma.

In this regard, CXCR3 expression is rapidly upregulated early during differentiation of Th1 cells under Ag stimulation whereas the cytokine milieu has a minimal impact (45, 46). As such, the inhibition of cytokine production by IL-10 and, particularly, IL-12 (47), is likely to play a minor role in CXCR3 expression while the reduced expression of MHC-II during priming, as seen in this study, may have a more significant contribution. However, we anticipate that other chemokine receptors may also contribute to the migration of Ag-specific CD4^+^ T cells into the lung parenchyma, particularly in high IL-10 environments. Specifically, the expression of CCR2 and CXCR5 (and, to a lesser degree, CCR5 and CXCR6) has recently been shown to participate in Th1 cell recruitment to the lungs of *M*. *tuberculosis*–infected mice (41). Particularly important is the expression of the chemokine CXCL13 (48, 49) and that of its receptor CXCR5 (17, 50); both are crucially required for T cells to efficiently migrate into the lung parenchyma and into the infected lesion. While we did not see a negative impact of IL-10 in CXCL13, we did see a reduced expression of CXCR5 as well as B cell areas in the lungs of pMT-10 mice (B6 = 7.79 ± 3.50 vs. pMT-10 = 0.76 ± 0.22, *P* ≤ 0.05).

The expression of indoleamine 2,3-dioxygenase (IDO1) has been shown to hamper the development of inducible bronchus-associated lymphoid tissue and to prevent the optimal homing of CD4^+^ T cells as well as their interaction with *M*. *tuberculosis*–infected phagocytes (51, 52). As the expression of IL-10 and that of IDO1 are linked (53), we questioned the impact of IDO1 in our model. However, we did not find increased expression of IDO1 in pMT-10 mice except at late time points after infection (Supplemental Figure 2). This suggests the activity of IDO1 is unlikely to contribute to the development of vasculature-associated CD4^+^ T cells seen in pMT-10 mice. On the other hand, IDO1 may contribute to the uncontrolled proliferation of *M*. *tuberculosis* at late stages of infection in pMT-10 mice with high levels of IFN-γ (Figure 2B) and with necrotic lesions (Figure 1C). However, this needs to be further addressed.

The developing consensus that protective CD4^+^ T cells locate in the lung parenchyma stems from studies showing that nonprotective antigen-specific T cells are associated with vasculature and express high KLRG1 and T-bet during chronic *M*. *tuberculosis* infection (16). KLRG1 expression marks a population of high cytokine-producing CD4^+^ T cells, particularly IFN-γ, during chronic *M*. *tuberculosis* infection, but not a self-renewing population. By contrast, PD-1 expression marks a population of self-renewing parenchymal CD4^+^ T cells (15). As the retention of CD4^+^ T cells in the vasculature of mice expressing high IL-10 is not a consequence of chronic antigen stimulation, we do not see increased KLRG1 expression. However, because progression of infection is associated with vasculature-associated CD4^+^ T cells, our data further support parenchymal migratory potential as a key feature of protective CD4^+^ T cells.

The fact that we see CD4^+^ T cells expanding in the lungs only after extensive bacterial proliferation occurs further reinforces the crucial role of the parenchymal migratory capacity of T cells for their response and protective function. As such, the data show that one of the crucial antagonistic effects of IL-10 in the control of *M*. *tuberculosis* infection is impeding the differentiation of effector CD4^+^ T cells with parenchymal migratory capacity. Accordingly, IL-10 overexpression after the onset of T cell responses does not influence the host’s overall bacterial burden or disease pathology. We saw reduced frequency of CD3^+^CD4^+^ T cells (B6 = 80.33 ± 4.07 vs. pMT-10 = 73.09 ± 4.52, *P* ≤ 0.01) and CD4^+^ T cells capable of producing IFN-γ in response to ESAT6_1–20_ (B6 = 3.47 ± 0.53 vs. pMT-10 = 2.9 ± 0.74, not significant) at day 65 after infection. However, this T cell reduction is likely a consequence of the IL-10 influence in the local environment of the lung lesion, as recently investigated (54). Moreover, it supports the limited contribution of IFN-γ–producing CD4^+^ T cells in the control of *M*. *tuberculosis* infection in the lungs (55).

Although we do not see a negative impact of IL-10 during chronic infection, we do not exclude the possibility that further extending the overexpression of IL-10 throughout chronic infection would result in increased bacterial burdens, as other studies have suggested (25, 33, 39, 54, 56). However, the most likely explanation for this discrepancy is that IL-10 overexpression in our model is controlled by zinc supplementation; indeed, the levels of IL-10 are normal before zinc supplementation whereas, in other models, IL-10 is likely overexpressed throughout infection (25, 33). As such, these models’ effects of IL-10 during the early stages of infection will affect bacterial burdens during chronic infection. Accordingly, the susceptible phenotype of CBA/J to *M*. *tuberculosis* infection is more clearly seen during chronic infection (24, 57); however, the blockade of IL-10 signaling during the first 21 days of infection was sufficient to revert the susceptible phenotype of CBA/J mice (26). These data further highlight the crucial role of IL-10 during the onset of the T cell response.

Previous data have shown that the early control of *M*. *tuberculosis* infection in BCG-vaccinated mice is not mediated by recently activated effectors, but likely by memory cells colonizing the lungs (58). Furthermore, blockade of IL-10R signaling during BCG vaccination has been shown to significantly increase protection against aerogenic challenge with *M*. *tuberculosis* (34). One key challenge in the rational design of new TB vaccines is to overcome the delayed activation and expression of antigen-specific responses in the lungs following aerosol challenge (59, 60). Therefore, together with results from earlier studies, our data suggest that targeting IL-10 or the IL-10 pathway during vaccination may help overcome the delayed T cell response and enhance vaccine-induced protection.

Together our data demonstrate that IL-10 antagonizes the control of pulmonary *M*. *tuberculosis* infection by impeding the differentiation of T cells with capacity to migrate into lung parenchyma. These data, when taken together with other recent data showing enhanced vaccine-induced protection through IL-10 blockade during BCG vaccination and after *M*. *tuberculosis* infection (34), suggest that IL-10 may be an impeding factor to the rapid expression of T cell immunity in the lungs. Future vaccines should promote the development of T cells capable of rapidly migrating, of persisting within the lung parenchyma, and of colocating with infected phagocytes.

## Methods

### Mice.

C57BL/6 and B6.129S7-Rag1^tm1Mom^/J (RAG^–/–^) mice were bred at the ICVS animal facility from stock purchased from Charles River Laboratories and the Jackson Laboratory, respectively. Breeders of P25 TCR transgenic mice that recognize Peptide-25 of the immunodominant antigen Ag85b of *M*. *tuberculosis*, presented in the context of I-A^b^, were originally obtained from Anne O’Garra’s lab at the Francis Crick Institute, London, United Kingdom; these were previously described (61). pMT-10 mice on a C57BL/6 background were produced by António G. Castro and Paulo Vieira, as previously described (35). Briefly, mouse IL-10 cDNA sequence was cloned in the p169ZT vector, which carries the sheep metallothionein (MT) Ia promotor, a β-globin splice site and the SV40 polyadenylation signal. The resulting vector was injected in C57BL/6 eggs and transgenic founders were identified by PCR using MT-specific primers. IL-10 overexpression in pMT-10 mice was induced by supplementing their drinking water with 2% sucrose solution containing 50 mM of zinc sulphate, as previously described (35). The resulting rapid increase in circulating levels of IL-10 were maintained until zinc sulphate was withdrawn (35). B6 mice used as controls were maintained in the same condition as pMT-10 mice, including drinking water supplemented with 2% sucrose solution containing 50 mM of zinc sulphate. Both male and female mice between the ages of 6 and 12 weeks were used for experimental procedures.

### M. tuberculosis aerosol infection and bacterial load determination.

The H37Rv strain of *M*. *tuberculosis*, originally from the Trudeau Institute, was used in this study. To generate *M*. *tuberculosis*–mCherry, the parental *M*. *tuberculosis* strain was transformed using the plasmid pBP10 provided by David G. Russel, Department of Microbiology and Immunology, College of Veterinary Medicine, Cornell University, Ithaca, New York, USA, as described previously (62, 63). Both *M*. *tuberculosis* and *M*. *tuberculosis*–mCherry were grown to log phase in Middlebrook 7H9 broth supplemented with 10% oleic acid/albumin/dextrose/catalase (OADC), 0.2% glycerol, 0.05% Tween 80, and 50 μg/ml hygromycin B (for *M*. *tuberculosis*–mCherry), and subcultured in Proskauer Beck medium with 0.05% Tween 80 to mid-log phase before frozen at −80°C. These frozen stocks were quantified and used to infect mice with a low dose of bacteria (~75 CFUs) using a Glas-Col airborne infection system, as previously described (18). At selected time points after challenge, mice were killed by CO_2_ asphyxiation; organs were aseptically excised and individually homogenized in saline. Organ homogenates were then 10-fold serial-diluted and plated on nutrient 7H11 agar (BD Biosciences) for 3 weeks at 37°C, at which point CFUs were counted.

### Sample collection and preparation of lung single-cell suspensions.

Aseptically excised lungs were sectioned and incubated at 37°C for 30 minutes with collagenase D (0.7 mg/mL, Sigma-Aldrich). Lungs were then disrupted into a single-cell suspension by passage through a 70 μm nylon cell strainer (BD Biosciences). After centrifugation, the cell-free suspensions were aliquoted and frozen at −80°C until their concentrations of cytokines were determined using ELISA kits (Thermo Fisher Scientific), following the manufacturer’s instructions. Lung single-cell suspensions were then treated with erythrocyte lysis buffer (0.87% of NH_4_Cl). To remove cell debris and nonhematopoietic cell interference, lung cells were further processed over a 40:80% Percoll (GE Healthcare). The resulting cell suspension was washed twice and counted. A single-cell suspension was prepared from the MLNs by passing the organs through a 70 μm nylon cell strainer (BD Biosciences), followed by treatment with erythrocyte lysis buffer.

For intravital flow cytometry analysis, mice received APC-labeled CD45 antibody intravenously 3 minutes before euthanasia (16). Lung or MLN single-cell suspensions were then stained with fluorochrome-conjugated antibodies for 30 minutes on ice. For tetramer staining, cells were incubated in the dark for 1 hour at 37°C with the Brilliant Violet 421–conjugated I-A^b^ ESAT-6_4–17_ tetramer provided by the NIH Tetramer Core Facility. For intracellular cytokine detection, cells were cultured in 5 μg/ml of ESAT-6_1–20_ peptide for 1.5 hours before 10 μg/ml Brefeldin A (Sigma-Aldrich) was added to the culture for an additional 3.5 hours. The cells were phenotyped using the following antibodies: CD3 Brilliant Violet 605 (145-2C11, BioLegend), CD4 APC-Cy7 (GK1.5, BioLegend), CD11b PE-Cy7 (M1/70, BioLegend), CD11c Brilliant Violet 650 (N418, BioLegend), Ly-6C PerCPCy5.5 (HK1.4, BioLegend), MHC-II FITC (M5/114.15.2, BioLegend), CD86 APC (GL-1, BioLegend), CD44 PerCPCy5.5 (IM7, BioLegend), CD69 PE (H1.2F3, eBioscience), CD62L PE/Cy7 (MEL-14, BioLegend), CD45 Brilliant Violet 510 (30-F11, BioLegend), CD45.2 APC (104, BioLegend), Ki-67 PE/Cy7 (SoIA15, eBioscience) and IFN-γ PE/Cy7 (XMG1.2, BioLegend). Data were acquired on an LSR II flow cytometer (BD Biosciences) using Diva software and analyzed using FlowJo software (BD Biosciences). The total number of cells for each population was determined based on the percentage of cells measured by flow cytometry and the total number of cells per lung.

### CD4^+^ T cell adoptive transfers.

For adoptive transfers of P25 TCR-Tg CD4^+^ T cells, the spleens of P25 mice were collected and processed into single-cell suspensions. Naive CD4^+^ T cells were negatively selected using the Naive CD4^+^ T Cell Isolation Kit from Miltenyi Biotec, following the manufacturer’s instructions. Purified cells were then stained with 5 μM CFSE (Molecular Probes) for 10 minutes at 37°C and adoptively transferred into recipient B6 and pMT-10 mice (1 × 10^6^ total CD4^+^ T cells per mouse). The following day, B6 and pMT-10 recipients were sacrificed and their MLNs analyzed for the presence of CFSE^+^ (P25TCR-tg) transferred cells.

For adoptive transfer of effector CD4^+^ T cells into RAG^–/–^ mice, the lungs of B6 and pMT-10 mice were harvested at day 28 after *M*. *tuberculosis* infection and processed into single-cell suspensions, as described above. CD4^+^ T cells were magnetically labeled with CD4 (L3T4) microbeads (Miltenyi Biotec) and purified following the manufacturer’s instructions. Positively selected lung CD4^+^ T cells were then counted and injected intravenously into RAG^–/–^ mice infected with *M*. *tuberculosis* 15 days earlier (5 × 10^5^ cells per mouse). At day 30 after infection (15 days after adoptive transfers), the lungs were aseptically excised to determine bacterial loads.

### Anti–IL-10R mAb treatment.

One day prior to *M*. *tuberculosis* infection, mice were injected i.p. with 1 mg of either anti–IL-10 receptor (IL-10R) mAb (CD210) or IgG1 isotype control mAb (both from Bio X Cell), as previously described (64). Mice were then infected with *M*. *tuberculosis* through the aerosol route. To maintain IL-10R blockade, mice received 0.35 mg of the respective mAb i.p. at weekly intervals until the designated experimental times.

### Histology and immunohistochemistry.

The upper right lobe of each lung was inflated with 4% PFA and processed routinely for light microscopy with hematoxylin and eosin stain. Morphometric analysis was performed in a blinded manner using ImageJ software (version 1.50e; NIH). The percentage of total lung area involved with inflammation was calculated by dividing the cumulative area of inflammation by the total lung surface area for each sample.

Immunofluorescence was performed on formalin-fixed lung sections as described previously (65). Sections were probed using a purified rabbit polyclonal anti-CD3e (1:100; ab185811, Abcam), and visualized by adding Alexa Fluor 568 goat anti-rabbit (1:500; A-11011, Invitrogen). SlowFade Gold Antifade Mountant with DAPI (Invitrogen) was used to counterstain tissues and to detect nuclei. Representative images were obtained using an Olympus BX61 microscope and were recorded using a digital camera (DP70, Olympus) using the Olympus cell^P software.

### Quantitative RT-PCR analysis.

Quantitative RT-PCR (qRT-PCR) was performed as previously described (66). Total RNA from whole lungs was extracted using TRIzol (Invitrogen) from which cDNA was generated using the GRS cDNA Synthesis Mastermix (Grisp), following the manufacturer’s instructions. The resultant cDNA template was used to quantify the expression of target genes by qRT-PCR (CFX96 Real-Time System with C1000 Thermal Cycler, Bio-Rad) and normalized to ubiquitin mRNA levels using the ΔCt method. Target gene mRNA expression was quantified using SYBR Green (Thermo Fisher Scientific) and specific oligonucleotides (Invitrogen).

### Statistics.

Differences between groups were analyzed using a 2-tailed unpaired Student’s *t* test or 1-way ANOVA as appropriate. Survival curves were analyzed using the log-rank test. Data were displayed as mean ± SD. Differences were considered significant when *P* ≤ 0.05.

### Study approval.

All procedures involving live animals were performed in accordance with the European Directive 86/609/EEC and approved by the Subcomissão de Ética para as Ciências da Vida e da Saúde (SECVS 074/2016) (University of Minho, Braga, Portugal) and the Portuguese National Authority Direcção Geral de Alimentação e Veterinária (DGAV 014072) (Lisbon, Portugal).

## Author contributions

CMF, AGC, and ET conceived and designed the study. CMF, AMB, and PBS performed the experimental work and data analysis. RS, CC, AC, FR, and MCN provided technical and/or material support. CMF, AGC, and ET drafted the manuscript. AGC and ET acquired funding. All authors critically revised and approved the manuscript and accepted accountability.

## Supplementary Material

Supplemental data

## Version 1 09/23/2021

In-Press Preview

## Version 2 11/08/2021

Electronic publication
