# Supplementary material for: Early IL-10 promotes vasculature-associated CD4+ T cells unable to control Mycobacterium tuberculosis infection
Source: JCI Insight. 2021 Nov 8;6(21):e150060. doi: 10.1172/jci.insight.150060 (PMC8663558; doi:10.1172/jci.insight.150060)
Supplement: Supplemental data [file jciinsight-6-150060-s088.pdf]

SUPPLEMENTAL FIGURES

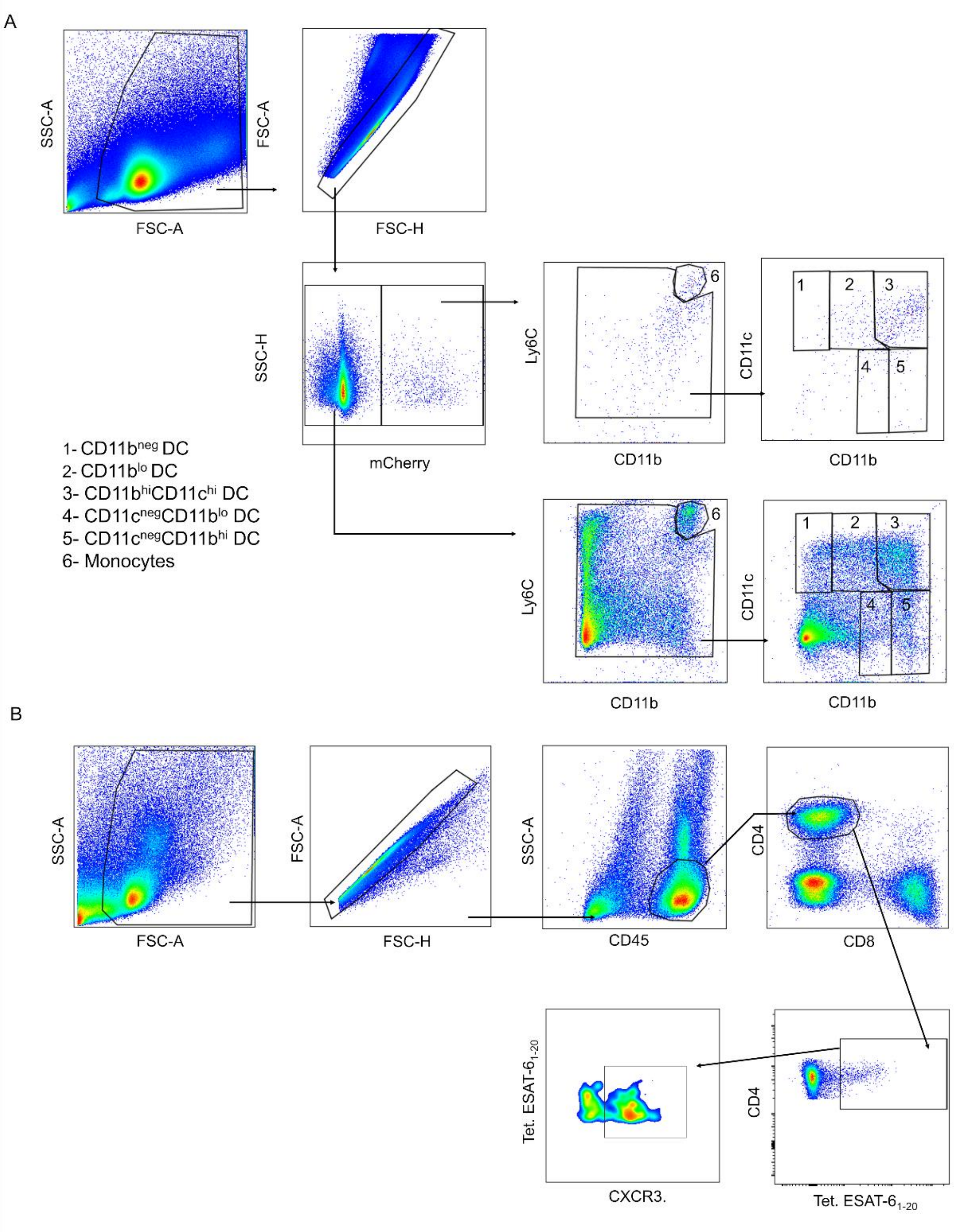

Supplemental Figure 1- Gating strategy used to identify (A) myeloid cell populations in the mLN, and (B) CXCR3 expression by I-A^{b} ESAT-6$_{4-17}$-specific CD4^{+} T cells in the lungs of Mtb-infected mice.

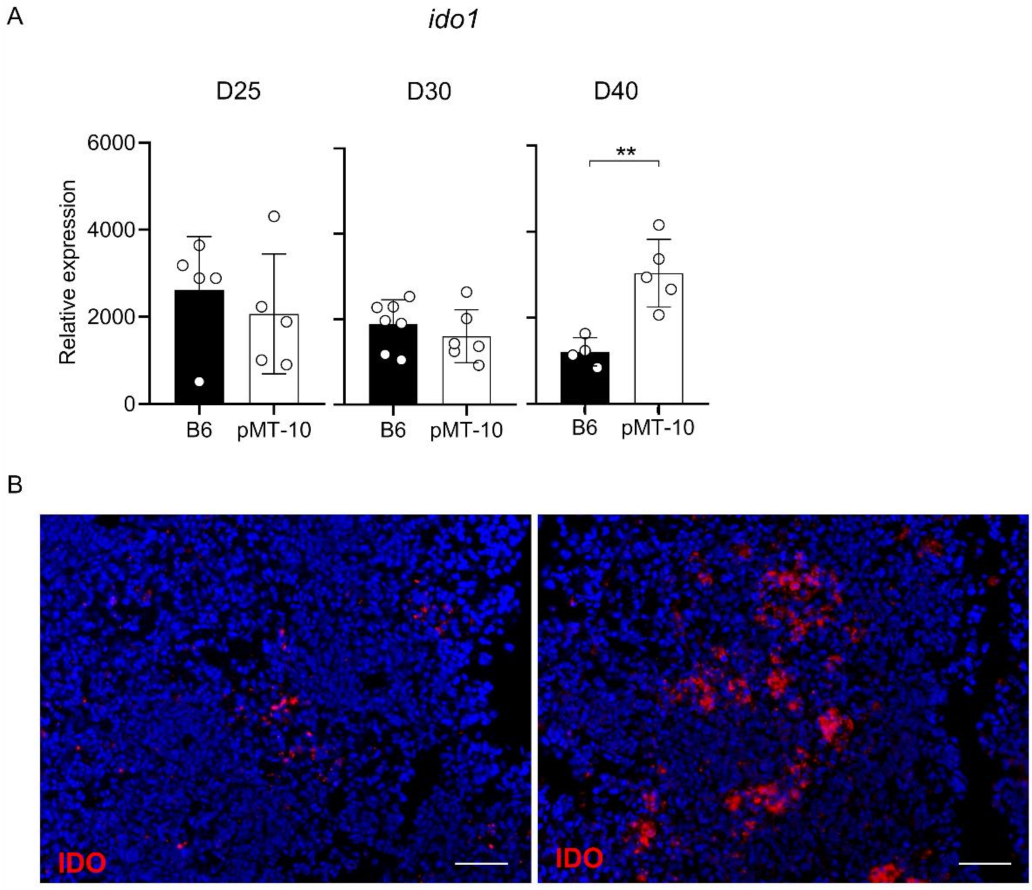

Supplemental Figure 2- Increased expression of indoleamine 2,3-dioxygenase (IDO1) in pMT-10 mice at the very late stages of Mtb infection. B6 and pMT-10 mice were infected with Mtb H37Rv via aerosol route and IL-10 overexpression was induced after day 5 post-infection. **(A)** Relative expression of *Ido1* in the lungs of infected mice at days 25, 30 and 40 post-infection. **(B)** Representative immunofluorescence staining of $IDO1^+$ cells in the lungs of mice at day 40 post-infection. Sections were probed with a purified rabbit polyclonal anti-IDO antibody (1:100; PA5-24598, ThermoFisher Scientific), and visualized by adding Alexa Fluor 568 goat anti-rabbit (1:500; A-11011, Invitrogen). Scale bar represents 50µm. Data are representative of 2 independent experiments with 4-5 mice per group. Error bars represent the mean ± SD. **, $P < 0.01$ using student's t-test.
